# Retraction: Anti-vibration method for sensing ring of fiber optical current transformer integrating structural design and adaptive signal processing

**DOI:** 10.1371/journal.pone.0352943

**Published:** 2026-07-06

**Authors:** The PLOS One Editors

## Notice of Republication

The *PLOS One* Editors retract this article [1] as it was erroneously published by PLOS, as a duplicate of [2,3]. The publisher apologizes for the error.

Additionally, the article [1] was republished on April 21, 2026, to address an issue identified post-publication.

The correct version of the article [4] is now available on the journal website.
